# Follow-Up CT Patterns of Residual Lung Abnormalities in Severe COVID-19 Pneumonia Survivors: A Multicenter Retrospective Study

**DOI:** 10.3390/tomography8030097

**Published:** 2022-04-20

**Authors:** Giulia Besutti, Filippo Monelli, Silvia Schirò, Francesca Milone, Marta Ottone, Lucia Spaggiari, Nicola Facciolongo, Carlo Salvarani, Stefania Croci, Pierpaolo Pattacini, Nicola Sverzellati

**Affiliations:** 1 Radiology Unit, Azienda USL-IRCCS di Reggio Emilia, 42123 Reggio Emilia, Italy; giulia.besutti@ausl.re.it (G.B.); lucia.spaggiari@ausl.re.it (L.S.); pierpaolo.pattacini@ausl.re.it (P.P.); 2 Department of Medical and Surgical Sciences, University of Modena and Reggio Emilia, 41121 Modena, Italy; 3 Clinical and Experimental Medicine PhD Program, University of Modena and Reggio Emilia, 41121 Modena, Italy; 4 Department of Medicine and Surgery (DiMec), University of Parma, 43126 Parma, Italy; silvischiro@gmail.com (S.S.); nicola.sverzellati@unipr.it (N.S.); 5 Unit of Scienze Radiologiche, University Hospital of Parma, 43126 Parma, Italy; cmanna@ao.pr.it; 6 Epidemiology Unit, Azienda USL-IRCCS di Reggio Emilia, 42122 Reggio Emilia, Italy; marta.ottone@ausl.re.it; 7 Respiratory Disease Unit, Azienda USL-IRCCS di Reggio Emilia, 42123 Reggio Emilia, Italy; nicola.facciolongo@ausl.re.it; 8 Rheumatology Unit, Azienda USL-IRCCS di Reggio Emilia, 42123 Reggio Emilia, Italy; carlo.salvarani@ausl.re.it; 9 Clinical Immunology, Allergy and Advanced Biotechnologies Unit, Azienda USL-IRCCS di Reggio Emilia, 42123 Reggio Emilia, Italy; stefania.croci@ausl.re.it

**Keywords:** COVID-19, follow-up, CT scan, fibrotic changes, CT patterns

## Abstract

Prior studies variably reported residual chest CT abnormalities after COVID-19. This study evaluates the CT patterns of residual abnormalities in severe COVID-19 pneumonia survivors. All consecutive COVID-19 survivors who received a CT scan 5–7 months after severe pneumonia in two Italian hospitals (Reggio Emilia and Parma) were enrolled. Individual CT findings were retrospectively collected and follow-up CT scans were categorized as: resolution, residual non-fibrotic abnormalities, or residual fibrotic abnormalities according to CT patterns classified following standard definitions and international guidelines. In 225/405 (55.6%) patients, follow-up CT scans were normal or barely normal, whereas in 152/405 (37.5%) and 18/405 (4.4%) patients, non-fibrotic and fibrotic abnormalities were respectively found, and 10/405 (2.5%) had post-ventilatory changes (cicatricial emphysema and bronchiectasis in the anterior regions of upper lobes). Among non-fibrotic changes, either barely visible (*n* = 110/152) or overt (*n* = 20/152) ground-glass opacities (GGO), resembling non-fibrotic nonspecific interstitial pneumonia (NSIP) with or without organizing pneumonia features, represented the most common findings. The most frequent fibrotic abnormalities were subpleural reticulation (15/18), traction bronchiectasis (16/18) and GGO (14/18), resembling a fibrotic NSIP pattern. When multiple timepoints were available until 12 months (*n* = 65), residual abnormalities extension decreased over time. NSIP, more frequently without fibrotic features, represents the most common CT appearance of post-severe COVID-19 pneumonia.

## 1. Introduction

As of April 2022, there are more than 490 million COVID-19 survivors all over the world [1]. Similar to what happened with previous endemic infections [2,3,4,5], residual lung abnormalities have been observed in follow-up CT scans of COVID-19 survivors.

Most data were obtained from short- and medium-term follow-up CT scans (median 3–6 months) [6,7,8,9,10,11,12,13,14]. Notably, residual CT lung abnormalities have been reported in 23–72% COVID-19 survivors 6 months after the disease [7,8,9,10,11,12,13]. Not surprisingly, ground glass opacities (GGO) represent the most common residual feature [7,8,9,10,11,12,13,14], while the frequency of CT features suggestive of lung fibrosis have been variously reported at 3 to 6 months, ranging from 1% to 70% [9,10,11,12,13].

Although further improvement was observed at subsequent follow-ups, both frequency data and characterization of post-COVID lung fibrosis at 12 months or later remain heterogeneous and controversial [13,15,16,17,18,19], mostly due to a lack of consistency in the interpretation of individual CT abnormalities.

Most previous studies recorded the frequency of individual CT abnormalities. This data could be insufficient to understand post-COVID interstitial lung disease. In fact, either reticulation or GGO could reflect different pathologic abnormalities, either fibrotic or non-fibrotic. Furthermore, the interpretation of established CT features of lung fibrosis (e.g., traction bronchiectasis) could also be questionable [20]. Hence, at present, it is still difficult to evaluate which findings represented CT features of permanent fibrotic lung disease or CT features of slowly resolving organizing pneumonia [21]. More information could come from the visual assessment of the CT patterns to improve the clinic-radiologic clustering of patients with post-COVID syndrome.

In this study, readers evaluated CT residual pulmonary abnormalities of patients who had severe COVID pneumonia as a whole, defining a comprehensive pattern.

## 2. Materials and Methods

### 2.1. Setting

The first COVID-19 pandemic wave in the Reggio Emilia and Parma provinces, two adjacent territories in Northern Italy, lasted from the end of February until May 2020. As of 15 May 2020, there had been 4863 and 4888 RT-PCR-confirmed COVID-19 cases in the province of Reggio Emilia and Parma, respectively.

### 2.2. Study Design and Ethics

This was a two-centers retrospective cohort study based on routinely collected data. The study was approved by the Area Vasta Emilia Nord (AVEN) Ethics Committee (protocol numbers 855/2020/OSS/AUSLRE and 1078/2020/OSS/AOUPR). Given the retrospective nature of the data collection, the Ethics Committee authorizes the use of a patient’s data without his/her informed consent if all reasonable efforts have been made to contact that patient to obtain it.

### 2.3. Study Population

All consecutive COVID-19 patients who underwent a follow-up CT scan 5–7 months after COVID-19 pneumonia diagnosis were enrolled. In one center (Azienda USL-IRCCS of Reggio Emilia), a routine 6–7-month follow-up CT scan was proposed to all COVID-19 survivors who had been hospitalized during the disease course with severe pneumonia (defined as respiratory failure during hospital stay, history of invasive or non-invasive mechanical ventilation and/or tocilizumab (TCZ) administration, or total extent of disease >40% at baseline CT), unless a first follow-up CT scan performed at 2–3 months revealed no residual lung abnormalities. Likewise, in the other center (Azienda Ospedaliero-Universitaria of Parma), a routine 5–7-month follow-up CT scan was considered for those subjects with radiologically and/or laboratory documented diagnosis of COVID-19-related pneumonia who had been previously admitted to the hospital and had a total extent of disease ≥30% at baseline CT evaluation and/or respiratory failure during hospitalization. Thus, the only exclusion criterion was the unavailability of 5–7-month follow-up chest CT scan due to patient refusal or complete imaging resolution at 2–3 months.

### 2.4. Clinical Data Collection

The COVID-19 Surveillance Registry, coordinated by the National Institute of Health and implemented in each Local Health Authority, was used to retrieve data on the dates of symptom onset, diagnosis, and hospitalization. Patients’ medical records were reviewed to collect data on comorbidities and COVID-19 treatment, symptoms, and mechanical ventilation (invasive and non-invasive). Blood tests at Emergency Department presentation were retrieved from the laboratory information system. The baseline visually estimated extent of disease was retrieved from the structured reports of baseline CT scans. COVID-19 disease course was defined as severe if respiratory failure occurred during hospitalization.

### 2.5. Follow-Up CT Scan

All the scans were HRCT images, acquired with the patient in the supine position during end-inspiration breath-hold, without intravenous contrast material administration. All technical data about the CT scans for both centers are reported in Appendix A.

For each center, two radiologists (GB and LS for Reggio Emilia, SS and NS for Parma, with 5 and 20 years of experience respectively) blinded to clinical data, independently and retrospectively reviewed the CT scans. Readings with divergent findings were discussed with the most experienced radiologist (NS).

Each reader classified the 5–7 month follow-up CT scans as follows: (1) resolved abnormalities, (2) residual non-fibrotic abnormalities, and (3) residual fibrotic abnormalities [22]. Absent or trivial residual CT abnormalities (e.g., subtle GGO and reticulations occupying <5% lung parenchyma) were both considered in keeping with resolution (complete or barely complete resolution).

Established CT features of residual fibrotic abnormalities included: subpleural reticular opacities, traction bronchiectasis, honeycombing, and signs of volume loss (e.g., fissural and broncho-vascular displacement). The dominant CT fibrotic pattern was assigned according to the classification of either idiopathic interstitial pneumonias or idiopathic pulmonary fibrosis [23,24]. Post-ventilatory abnormalities were considered when cystic spaces were visible in the subpleural interface of the anterior part of the upper and middle lobes [25].

In the absence of CT fibrotic features, individual CT abnormalities were retained as compatible with non-fibrotic CT abnormalities. These included: GGO, the ‘tinted sign’ [20], consolidation, perilobular opacities, parenchymal bands, nodules, and bronchial distortion. The dominant CT pattern was classified as follows: non-fibrotic non-specific interstitial pneumonia (NSIP), organizing pneumonia (OP), and mixed NSIP-OP. Both the global extent (to the nearest 5%) and distribution of individual pulmonary abnormalities were assessed (%).

In order to evaluate the longitudinal behavior of any residual pulmonary disease, each CT timepoint was compared with all the available previous CT timepoints (e.g., when available, ≥11-month CT timepoint was compared with 6-month, 3-month, and baseline CT timepoints).

### 2.6. Statistical Analyses

Clinical data and imaging findings were reported as median (interquartile range—IQR) for continuous variables and numbers and percentages (%) for categorical variables. For the cohort of Reggio Emilia, the inter-rater agreement for CT patterns and individual CT findings was calculated between the two local radiologists with low and high experience (GB and LS, respectively), and between the two local radiologists and the most experienced reader (NS). The kappa-statistic measure of weighted inter-rater agreement was used.

## 3. Results

### 3.1. Study Population

**Figure 1:**
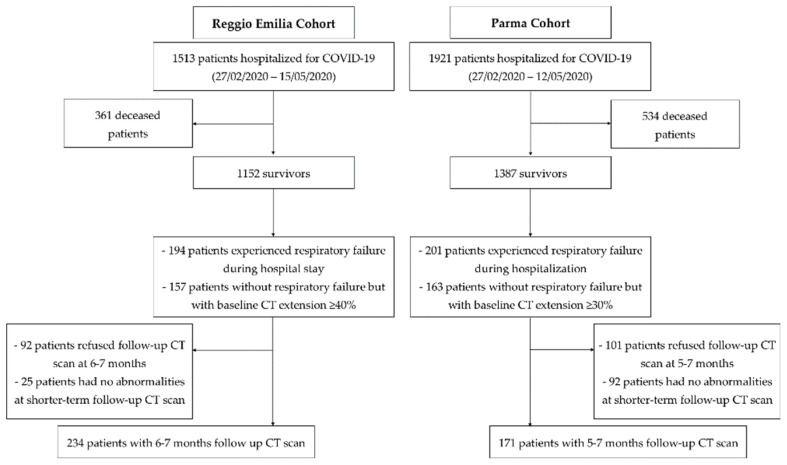
Flow chart depicting patient enrollment for the two study cohorts.

A total of 405 patients—234 (57.8%) and 171 (42.2%) from the Reggio Emilia and Parma hospitals, respectively—underwent a follow-up CT scan at 5–7 months from disease diagnosis, and were included in the present study (Figure 1). Demographic and clinical data of patients included in the two study cohorts are summarized in Table 1. The two cohorts were similar in terms of age, gender proportion, and comorbidity frequency. Initial symptoms were proportionally similar in the two cohorts, even if fever and cough were slightly more frequent in the Reggio Emilia cohort and dyspnea in the Parma cohort. Mean O_2_ saturation at admission was around 92% in both cohorts, and laboratory data at admission were only slightly different; for example, CRP levels were moderately higher in the Parma cohort. Median baseline CT extension was 30% and 50% for the Parma and Reggio Emilia cohorts, respectively. The proportion of clinically severe disease during hospitalization was similar.

### 3.2. 5–7-Month Follow-Up CT Scan

Complete or barely complete resolution of lung abnormalities was observed in 225 (55.6%) patients (Table 2).

**Figure 2:**
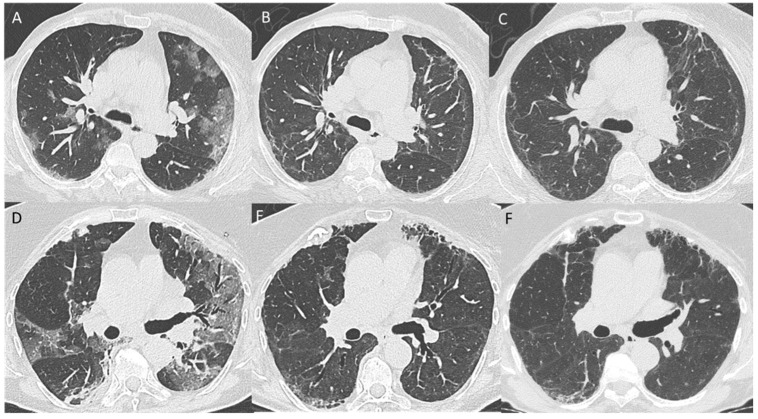
Baseline (**A**), 6-month follow-up (**B**), and 12-month follow-up (**C**) axial CT images showing the evolution of organizing pneumonia (OP) features towards residual non-fibrotic abnormalities resembling NSIP. Baseline (**D**), 6-month follow up (**E**), and 12-month follow-up (**F**) axial CT scans, showing patchy ground glass opacities (GGO) that are progressively replaced by reticular abnormalities and mild traction bronchiectasis resembling a fibrotic NSIP pattern.

Residual non-fibrotic abnormalities (Figure 2) were found in 152 (37.5%) patients. Among these abnormalities, overt GGO was relatively rare (4.9% of the whole population), while barely visible GGO was found in 27.2% of the whole population. Bronchiectasis, predominantly peripheral in distribution, was described in 12.8% patients, and perilobular opacities in 7.9% patients, while other findings, such as parenchymal bands and consolidations, were rarer (<3%). When possible, a predominant pattern was assigned to the 152 patients with residual non-fibrotic abnormalities, resulting in non-fibrotic NSIP as the most frequent pattern (103/152), followed by mixed NSIP-OP pattern (32/152) and OP pattern (12/152).

Residual fibrotic abnormalities (Figure 2) were found in 28 (6.9%) patients (Table 2). Ten out of 28 (2.5% of the whole cohort) were classified as post-ventilatory fibrotic abnormalities. These post-ventilatory abnormalities were found in 10/43 (23.3%) patients who received invasive mechanical ventilation. The remaining 18/28 patients with residual fibrotic abnormalities were classified mostly as non-fibrotic NSIP (14/18), while UIP and probable UIP were found in 1/18 and 2/18 patients. Honeycombing was visible in 3 patients only (0.7% of the whole cohort), one of them secondary to ventilatory damage. Traction bronchiectasis, more frequently peripheral in distribution and mild to moderate in severity, was found in 16/18 (88.9%) patients with residual fibrotic changes and 9/10 (90%) patients with post-ventilatory abnormalities. Subpleural reticulations and GGO were visible in 15/18 (83.3%) and 14/18 (77.8%) patients with residual fibrotic abnormalities, and in 7/10 (70%) and 9/10 (90%) patients with post-ventilatory abnormalities, respectively. Cicatricial emphysema was visible in the anterior region for the upper lobes in 6/10 (60%) patients with post-ventilatory abnormalities.

The visually-estimated global extension of parenchymal abnormalities was higher for fibrotic and post-ventilatory abnormalities (median 30% and 45%, respectively) compared to non-fibrotic residual abnormalities (median 20%).

Interrater agreement was evaluated on the cohort of Reggio Emilia (Appendix B and Table A1).

### 3.3. Follow Up CT Findings: Comparison between the Two Cohorts

CT evolution patterns at 5–7 months had similar frequency rates in the two cohorts. The frequency of complete or barely complete resolution was 99/171 (57.9%) in the cohort from Parma and 126/234 (53.8%) in the cohort from Reggio Emilia, while residual non-fibrotic abnormalities were found in 61/171 (35.7%) patients of the Parma cohort and in 91/234 (38.9%) patients of the Reggio Emilia cohort. Finally, the rates of residual fibrotic abnormalities were 17/234 (7.2%) and 11/171 (6.4%) in the Reggio Emilia and Parma cohorts, respectively. Some differences between the two cohorts can be found in the individual CT findings of patients with residual non-fibrotic abnormalities, with the cohort from Reggio Emilia presenting higher rates of overt GGO (8.5% vs. 0%) and bronchiectasis (20.5% vs. 2.3%). The patterns of residual abnormalities were similarly distributed in the two cohorts, with the most prevalent patterns being non-fibrotic NSIP (approximately 25% in both cohorts) among non-fibrotic residual abnormalities, and fibrotic NSIP (approximately 3–4% in the two cohorts) among fibrotic residual abnormalities. Median global disease extension was similar in the two cohorts, only slightly lower in the Parma cohort (median 15% vs. 20% in the Reggio Emilia cohort for non-fibrotic residual abnormalities, and median 30% vs. 35% in the Reggio Emilia cohort for fibrotic abnormalities).

### 3.4. Pattern Evolution

**Figure 3:**
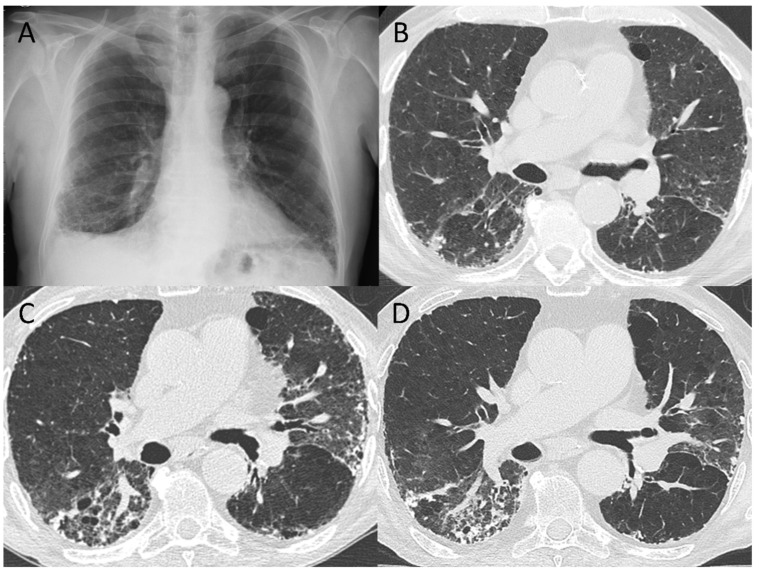
Representative images of a patient who had initial signs of interstitial lung disease at the lower lung zones in a chest X-ray (**A**) performed 2 years earlier than the baseline CT scan at COVID-19 diagnosis (**B**). In the 3-month (**C**) and 6-month (**D**) follow-up CTs the pattern was classified as residual fibrotic changes, possibly in keeping with COVID-19-induced progression of a pre-existing interstitial lung disease.

When available, multiple CT timepoints were evaluated. A CT scan performed 2–3 months after COVID-19 diagnosis was available for 23 out of the 28 (82%) patients who had residual fibrotic abnormalities at 5–7 months. A decrease in parenchymal extension was found over time. Two of these patients had signs of preexisting interstitial lung disease visible on their baseline CT scan (Figure 3). Conversely, in two cases, abnormalities that were originally classified as potentially fibrotic at 2–3 months (by the most unexperienced reader) were completely resolved at 6–7 months.

**Figure 4:**
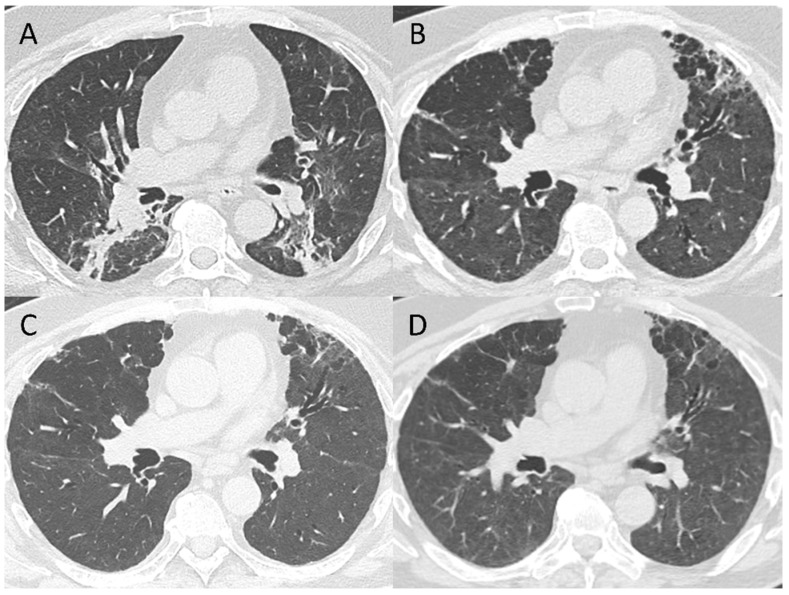
Representative CT images of post-ventilatory residual fibrotic abnormalities in a patient who received invasive mechanical ventilation. From baseline (**A**) to 3-month follow-up CT scan (**B**) a progressive resolution of GGO and consolidations at lower lobes can be observed, together with the appearance of bronchiectasis, GGO, and cystic spaces in the subpleural interface of the anterior part of the upper left lobe in keeping with post-ventilatory damage. These abnormalities are persistent at 6-month (**C**) and 12-month follow-up CT scans, even if a decrease in residual disease extension can be observed (**D**).

For a subset of 72 patients, a > 11 months follow-up CT scan was available, but 7/72 were excluded because of the occurrence of oncologic disease. Of the remaining 65 patients, 9 had only trivial residual abnormalities at 6–7 months, with complete resolution at 12 months; 46 had non-fibrotic residual abnormalities that in 26/46 cases completely resolved and in 20/46 cases decreased in extension but remained visible. Finally, the remaining 10 patients with fibrotic changes at 6–7 months (6 purely fibrotic and 4 post-ventilatory) had unchanged CT scans at 12 months, with the exception of a further slight decrease of global extension of residual abnormalities (Figure 4).

## 4. Discussion

In this study, we used a visual assessment of serial follow-up CT to characterize the CT patterns of residual pulmonary abnormalities in severe COVID-19 pneumonia survivors. By using this approach, at 5–7 month follow-up CT scan, a complete or barely complete resolution was observed in more than 55% patients, while 37.5% patients presented residual non-fibrotic abnormalities, and only 7% patients had residual fibrotic abnormalities (4.4% purely fibrotic and 2.5% post-ventilatory).

In previous studies, the heterogeneous definition of fibrotic changes led to a very wide range of reported residual fibrotic abnormalities rate. In previous studies evaluating follow-up CT scans at 6–7 months, the frequency of fibrotic-like changes ranged from 29% to 70%. The CT features of lung fibrosis—including parenchymal bands, reticular pattern, honeycombing, and traction bronchiectasis—were also variably reported [9,10,11]. Conversely, in a larger study of 353 patients, reticular pattern and interlobular septal thickening were rare (1%) at the 6-month follow-up CT scan [12]. Likewise, Wu et al. reported reticular opacities in 13 (16%) and bronchiectasis in only 1 of the 83 patients who underwent serial follow-up CT scans [13].

Similarly, the reported prevalence of residual fibrotic-like changes at the 12-month follow-up CT scan is variable. In a recent study of 209 survivors, 25% patients still had abnormalities at 12 months, including linear opacities and reticular/cystic abnormalities in 12% and 13% patients, respectively [17]. In a study of 94 patients, fibrotic lesions were described in 14% of patients at 12 months [18], while Han et al. reported that all the 40 patients found with fibrotic-like changes at 6 months (35% of the original cohort of 114 patients) still had fibrotic-like changes at 12 months [19]. Conversely, in the cohort of 83 patients described by Wu et al., changes such as interlobular septal thickening, reticular opacities, and subpleural curvilinear abnormalities were rare (5%) at 12 months, and none of the included patients had evidence of established fibrosis or progressive interstitial changes [13].

These studies mostly defined the presence of fibrotic changes based on individual CT findings such as reticulations, bands, and bronchiectasis. However, it has recently been argued that those fibrotic-like changes, in the setting of recovery from an acute lung injury, may have a completely different significance than in the setting of chronic lung diseases [22,26]. Bands probably represent limited organizing pneumonia, GGO that reduces in extension or attenuation may reflect inflammation and immature fibrosis remodeling, and bronchial distortion and traction bronchiectasis may not be irreversible (as previously described in SARS pneumonia follow-up CT scans) [20,21,22,27]. These hypotheses are in keeping with the progressive reduction of lung residual changes over time, even from 6 to 12 month follow-up CT scans, as shown in this study and by the other recent literature [13,15,16,17,18,19,28]. These findings may improve the understanding of the natural history of the disease regarding the radiologic evolution of COVID-19 infection, thus allowing a better targeting of therapeutic interventions through a multidisciplinary approach.

Given the variable interpretation of individual CT findings, the evaluation of global patterns rather than individual findings may improve the classification of residual abnormalities. By using this kind of approach, we found that only 7% patients had residual fibrotic changes, more than one third of them classified as post-ventilatory damage. The agreement between readers with different levels of experience was higher for the classification in CT patterns than for individual CT findings.

A major limitation of this study was the lack of information on symptoms and pulmonary function tests. However, this study focused on the interpretation of CT residual changes, and the correlation with symptoms and functional abnormalities was out of the scope of the present study. Moreover, the data on 12-month follow-up CT scans were limited to a subset of patients. Thus, the clinical significance of residual CT abnormalities is unknown, and the rate of residual changes at 1 year follow-up is uncertain. For this reason, the use of the term “fibrotic” may be inappropriate for those patients who may actually benefit from an improvement over a timespan longer than our follow-up. On the other hand, we lack data on the longer-term evolution of non-fibrotic findings, such as bands or linear opacities, which may persist for years after the disease.

In conclusion, by evaluating serial follow-up CT scans of 405 severe COVID-19 pneumonia survivors from two different institutions, we found a high rate (45%) of residual disease at 5–7 months after diagnosis, but only 7% patients had residual fibrotic abnormalities (4.4% purely fibrotic and 2.5% post-ventilatory), suggesting slow resolution rather than persistent fibrotic changes. NSIP was the most common pattern of residual abnormalities and was characterized primarily by the presence of barley visible GGO which was present in 72.3% of patients with residual non-fibrotic abnormalities. The evaluation of the evolution pattern rather than individual findings may improve the interpretation of residual lung changes after COVID-19 pneumonia.

## Figures and Tables

**Table 1:** Demographic and clinical characteristics of the included patients for the two cohorts. Continuous data are expressed as mean ± SD or median (IQR, interquartile range). Categorical data are presented as numbers with percentages in parentheses.

	Reggio Emilia Cohort Patients (*n* = 234)	Parma Cohort Patients (*n* = 171)
Female Sex, *n* (%)	70 (30%)	56 (33%)
Age, mean ± SD (years)	65.5 ± 11.1	63.6 ± 12.2
Comorbidities (at least one), *n* (%)	152 (65%)	120 (70%)
**Initial Symptoms**		
Fever	192 (82%)	118 (69%)
Cough	134 (57%)	68 (40%)
Dyspnea	63 (27%)	64 (37%)
Fatigue	15 (6%)	11 (6%)
Other Symptoms	60 (26%)	25 (15%)
Missing information	13 (6%)	37 (22%)
**Laboratory and CT data at admission**		
SatO_2_, mean (±SD) (%)	92.5 ± 4.7	91.6 ± 6
C-reactive protein (CRP), mean ±SD (mg/dL)	10.3 ± 7.6	13.6 ±7.3
PO_2_, mean ± SD (mmHg)	65.9 ± 14	75.1 ± 31
White blood cells count, mean ± SD (10^9^/L)	6.8 ± 5.3	7.6 ± 4
Lymphocytes percentage, mean ± SD (%)	20.5 ± 36.3	28.7 ± 20
CT disease extension at admission, median (IQR) (%)	50 (30; 55)	30 (15; 50)
**Disease severity**		
Non-severe, *n* (%)	79 (34%)	63 (37%)
Severe, *n* (%)	155 (66%)	108 (63%)

**Table 2:** 5–7-month follow-up CT scan patterns and individual abnormalities in the whole population and in the two cohorts separately. IQR, Interquartile range; GGO, ground glass opacities; NSIP, nonspecific interstitial pneumonia; OP, organizing pneumonia; UIP, usual interstitial pneumonia.

					Whole Population (*n* = 405)	Reggio Emilia Cohort (*n* = 234)	Parma Cohort (*n* = 171)
**Resolution**					225 (55.6%)	126 (53.8%)	99 (57.9%)
**Residual non-fibrotic abnormalities**					152 (37.5%)	91 (38.9%)	61 (35.7%)
	Global extension (%), median (IQR)				20% (10%; 30%)	20% (15%; 30%)	15% (5%; 30%)
	Overt GGO				20 (4.9%)	20 (8.5%)	-
	Barely visible GGO				110 (27.2%)	69 (29.5%)	41 (24.0%)
	Number of lobes involved by GGO, median (IQR)				4 (3; 5)	4 (3; 5)	3 (2; 5)
	Parenchymal bands				11 (2.7%)	7 (3.0%)	4 (2.3%)
				Lobar	-	-	-
				Peripheral	11 (2.7%)	7 (3.0%)	4 (2.3%)
	Consolidations				4 (1.0%)	4 (1.7%)	-
				Lobar		-	-
				Peripheral		4 (1.7%)	-
	Perilobular opacities				32 (7.9%)	25 (10.7%)	7 (4.0%)
	Nodules				2 (0.5%)	2 (0.9%)	-
	Bronchiectasis				52 (12.8%)	48 (20.5%)	4 (2.3%)
				Central	1 (0.2%)	1 (0.4%)	-
				Peripheral	44 (10.9%)	40 (17.1%)	4 (2.3%)
				Both	7 (1.7%)	7 (3.0%)	-
	Pattern			OP	12 (3.0%)	2 (0.9%)	10 (5.8%)
				Non-fibrotic NSIP	103 (25.4%)	60 (25.6%)	43 (25.1%)
				Mixed	32 (7.9%)	25 (10.7%)	7 (4.0%)
**Residual fibrotic abnormalities**					18 (4.4%)	11 (4.7%)	7 (4.0%)
		Global extension (%), median (IQR)			30% (20%; 39%)	30% (20%; 37.5%)	25% (20%; 50%)
		Subpleural reticulations			15 (3.7%)	10 (4.3%)	5 (2.9%)
		Bronchiectasis			16 (4.0%)	9 (3.8%)	7 (4.0%)
				Central	-	-	-
				Peripheral	12 (3.0%)	5 (2.1%)	7 (4.0%)
				Both	4 (1.0%)	4 (1.7%)	-
				Mild	8 (2.0%)	4 (1.7%)	4 (2.3%)
				Moderate	8 (2.0%)	5 (2.1%)	3 (1.8%)
				Severe	-	-	-
		Honeycombing			2 (0.5%)	1 (0.4%)	1 (0.6%)
		Volume loss			9 (2.2%)	5 (2.1%)	4 (2.3%)
		Ground glass opacities			14 (3.5%)	8 (3.4%)	6 (3.5%)
		Pattern	Fibrotic NSIP		14 (3.5%)	9 (3.8%)	5 (2.9%)
			UIP		1 (0.2%)	0 (0.0%)	1 (0.6%)
			UIP probable		3 (0.7%)	2 (0.9%)	1 (0.6%)
**Post-ventilatory abnormalities**					10 (2.5%)	6 (2.6%)	4 (2.3%)
		Global extension (%), median (IQR)			45% (32.5–60%)	45% (32.5–57.5%)	60% (35–84%)
		Subpleural reticulations			7 (1.7%)	6 (2.6%)	1 (0.6%)
		Bronchiectasis			9 (2.2%)	6 (2.6%)	3 (1.8%)
			Central		3 (0.7%)	3 (1.3%)	-
			Peripheral		5 (1.2%)	2 (0.9%)	3 (1.8%)
			Both		1 (0.2%)	1 (0.4%)	-
			Mild		3 (0.7%)	1 (0.4%)	2 (1.2%)
			Moderate		5 (1.2%)	5 (2.1%)	-
			Severe		1 (0.2%)	-	1 (0.6%)
		Honeycombing			1 (0.2%)	-	1 (0.6%)
		Volume loss			5 (1.2%)	3 (1.3%)	2 (1.2%)
		Ground glass opacities			9 (2.2%)	5 (2.1%)	4 (2.3%)
		Cicatricial emphysema			6 (1.5%)	4 (1.7%)	2 (1.2%)

## Data Availability

Participant data that underlie the results reported in this manuscript will be shared after de-identification, beginning 6 months and ending at least 7 years after article publication, to researchers who provide a methodologically sound proposal with objectives consistent with those of the original study. Proposals and data access requests should be directed to the Area Vasta Emilia Nord (AVEN) Ethics Committee at CEReggioemilia@ausl.re.it as well as to the Authors at the Epidemiology Unit of AUSL–IRCCS di Reggio Emilia at info.epi@ausl.re.it, who are the data guardians. To gain access, data requestors will need to sign a data access agreement.

## Appendix A. Methods-CT Scan Technical Data

### Appendix A.1. Reggio Emilia

Follow-up CT scans were performed using a 128-slice scanner (Somatom Definition Edge, Siemens Healthineers, Erlangen, Germany), without contrast media injection, with the patient in the supine position during end-inspiration. Scanning parameters were tube voltage 120 kV, automatic tube current modulation, collimation width 0.625 or 1.25 mm, acquisition slice thickness 2.5 mm, and interval 1.25 mm. Images were reconstructed with a high-resolution algorithm at slice thickness 1.0/1.25 mm.

### Appendix A.2. Parma

In Parma, follow-up CT scans were acquired with a 128-slice scanner (SOMATOM Definition Edge, Siemens Healthineers, Erlangen, Germany). All the scans were HRCT images, acquired with the patient in the supine position during end-inspiration breath-hold, without intravenous contrast material. The acquisition parameters were set at 110–120 kVp, 80 reference mAs, pitch 0.9–1.2, and collimation 0.625–1.0 mm. Reconstruction parameters for lung images: slice thickness 1.0 mm, increment 0.7–1.0 mm, sharp reconstruction algorithm (Bl57 or B70s, respectively), and lung window (width, 1600 HU; level, −600 HU). Reconstruction parameters for mediastinal images: slice thickness 2.0 mm, increment 1.5 mm, medium reconstruction algorithm (Br36 or B31s, respectively), and mediastinal window (width, 400 HU; level, 30 HU). Sinogram affirmed iterative reconstruction (SAFIRE) strength 3 on Definition Edge scanner.

## Appendix B. Inter-Rater Agreement

Inter-rater agreement, calculated on the cohort of patients from Reggio Emilia, was high for the classification of evolution pattern-resolution, residual fibrotic, and residual non-fibrotic abnormalities (k between 0.90 and 0.93 for different couples of readers). For individual CT findings, the inter-rater agreement was variable—lower for overt GGO, parenchymal bands, bronchiectasis, subpleural reticulation, honeycombing, and volume loss—for the comparisons involving the least experienced reader (k between 0.40 and 0.68) (Table A1).

**Table A1:** Inter-rater agreement.

	Reader 1-Reader 2		Reader 2-Reader 3		Reader 1-Reader 3	
	Kappa	*p*	Kappa	*p*	Kappa	*p*
CT pattern (resolution/residual non-fibrotic/residual fibrotic)	0.93	<0.0001	0.90	<0.0001	0.90	<0.0001
Global extension	0.98	<0.0001	0.98	<0.0001	0.96	<0.0001
Overt GGO	0.67	<0.0001	0.86	<0.0001	0.68	<0.0001
Barely visible GGO	0.98	<0.0001	0.97	<0.0001	0.97	<0.0001
Number of lobes involved by GGO	0.88	<0.0001	0.93	<0.0001	0.87	<0.0001
Parenchymal bands	0.61	<0.0001	0.72	<0.0001	0.66	<0.0001
Consolidations	0.75	<0.0001	0.86	<0.0001	0.89	<0.0001
Perilobular opacities	0.72	<0.0001	0.86	<0.0001	0.86	<0.0001
Bronchiectasis	0.67	<0.0001	0.85	<0.0001	0.72	<0.0001
Pattern of non-fibrotic abnormalities	0.99	<0.0001	0.98	<0.0001	0.97	<0.0001
Subpleural reticulation	0.68	<0.0001	0.83	<0.0001	0.74	<0.0001
Honeycombing	0.40	<0.0001	1.00	<0.0001	0.40	<0.0001
Volume loss	0.50	<0.0001	0.79	<0.0001	0.51	<0.0001
Cicatricial emphysema	1.00	<0.0001	1.00	<0.0001	1.00	<0.0001

Table A1. Inter-rater agreement in the assessment of CT patterns and individual findings. Reader 1 (GB) is the most unexperienced reader, Reader 2 (LS) has intermediate experience, while Reader 3 (NS) is the most experienced reader. GGO, ground glass opacities.
